# Antiproliferative effect of urolithin A, the ellagic acid-derived colonic metabolite, on hepatocellular carcinoma HepG2.2.15 cells by targeting Lin28a/let-7a axis

**DOI:** 10.1590/1414-431X20187220

**Published:** 2018-05-07

**Authors:** Zhenpeng Qiu, Junxuan Zhou, Cong Zhang, Ye Cheng, Junjie Hu, Guohua Zheng

**Affiliations:** 1 College of Pharmacy, Hubei University of Chinese Medicine, Wuhan, China; 2 Hubei Engineering Research Center of Viral Vector, Wuhan Institute of Bioengineering, Wuhan, China; 3 Key Laboratory of Chinese Medicine Resource and Compound Prescription, Ministry of Education, Hubei University of Chinese Medicine, Wuhan, China

**Keywords:** Urolithin A, Hepatocellular carcinoma, Lin28a, let-7a, Cell proliferation, HBx

## Abstract

An abnormality in the Lin28/let-7a axis is relevant to the progression of hepatitis B virus (HBV)-positive hepatocellular carcinoma (HCC), which could be a novel therapeutic target for this malignant tumor. The present study aimed to investigate the antiproliferative and anti-invasive effects of urolithin A in a stable full-length HBV gene integrated cell line HepG2.2.15 using CCK-8 and transwell assays. The RNA and protein expressions of targets were assessed by quantitative PCR and western blot, respectively. Results revealed that urolithin A induced cytotoxicity in HepG2.2.15 cells, which was accompanied by the cleavage of caspase-3 protein and down-regulation of Bcl-2/Bax ratio. Moreover, urolithin A suppressed the protein expressions of Sp-1, Lin28a, and Zcchc11, and elevated the expression of microRNA let-7a. Importantly, urolithin A also regulated the Lin28a/let-7a axis in transient HBx-transfected HCC HepG2 cells. Furthermore, urolithin A decelerated the HepG2.2.15 cell invasion, which was involved in suppressing the let-7a downstream factors HMGA2 and K-ras. These findings indicated that urolithin A exerted the antiproliferative effect by regulating the Lin28a/let-7a axis and may be a potential supplement for HBV-infected HCC therapy.

## Introduction

Liver cancer is one of the most malignant tumors globally, with high mortality rates, which commonly result from chronic inflammation and hepatic fibrosis (1). In high-incidence areas of hepatocellular carcinoma (HCC), such as West Africa approximately, 60% of HCC patients are infected with hepatitis B virus (HBV), suggesting that integrated HBV genetic material in hepatocytes may accelerate the process of cirrhosis and cell immortalization (2). Signaling pathways that activate cell proliferation and invasion are the effectors of HBx, a reverse transcription element in the HBV genome for fibrosis and oncogenesis, interacted by TGF-β or other transcriptional factors, such as nuclear factor-κB and Sp-1 (3–5). Consequently, although HBV replication in infected hepatocytes cannot be eradicated, acting on the HBx-triggered molecular networks and suppressing the cofactors of HBx might be a potent therapeutic strategy for HBV-infected HCC.

Dysregulated microRNA (miRNA) profiles are the collaborators of Hbx, which aggravate tumor progression involved in carcinogenesis, cell invasion, and metastasis in human malignancies. Let-7a, one of the let-7 family members, was initially characterized in *Caenorhabditis elegans* with Lin28a, an RNA-binding protein that acted as a suppressor of let-7 expression and controller for development and differentiation (6). Let-7a is also down-regulated in HCC patients and its low expression in liver tissues may contribute to poor survival rates (7). Moreover, Lin28-induced cancer cell EMT is dependent on the low let-7 level and overexpression of the EMT-associated let-7a downstream targets, such as K-ras and HMGA2 (8). Therefore, seeking an approach to alter the Lin28a/let-7a axis in hepatoma cancer cells may lead to the development of effective strategies for HCC therapy.

Urolithins, the dibenzopyran-6-one colonic metabolites derived from ellagic acid (EA) or ellagitannins (ETs), have been suggested to be beneficial for human health. Urolithins in target tissues and cells could act on sub-cellular components and activate signaling transduction. These cell responses contribute to the various biological potentials of EA- and ET-rich diets (9). Based on the anti-proliferative effects of urolithin A on HepG2 cells in our previous findings (10), the function of urolithin A in repressing HepG2.2.15 (HBV-integrated HepG2 cell line) cell proliferation and invasion are discussed in the present study. The results suggested that the regulating effects of urolithin A on the Lin28a/let-7a axis contributed to the inhibition of transcriptional factor Sp-1 and down-regulation of HMGA2 and K-ras in HepG2.2.15 cells.

## Material and Methods

### Chemicals

Urolithin A was synthesized as previously described (11). The purity (>94%) of urolithin A was evaluated by HPLC, and its molecular weight was confirmed by mass spectrometry analysis (Figure S1). A hydro-soluble tetrazolium salt WST-8 (2-(2-methoxy-4-nitrophenyl)-3-(4-nitrophenyl)-5-(2,4-disulfophenyl)-2H-tetrazolium, monosodium salt) was obtained from Dojindo Laboratories (Japan) for CCK-8 assay. All other chemicals and reagents were of analytical grade.

### Cell culture

HepG2 and HepG2.2.15 cells (HepG2 cells integrated with a stable wild-type full-length HBV genome) were obtained from American Type Culture Collection and cultured in Dulbecco's Modified Eagle Medium (Gibco, USA) supplemented with fetal bovine serum (10%), penicillin (1 mM) and streptomycin (1 mM) at 37°C in a humidified 5% CO_2_ atmosphere.

### Cell viability assay

The effect of urolithin A on HepG2.2.15 cell viability was evaluated using the CCK-8 assay with WST-8 according to manufacturer's protocol (12). Cell viability values were normalized as follows: [Final absorbance (urolithin A) / Final absorbance (control)] × 100%. IC_50_ value for HepG2.2.15 cells was calculated according to a dose-response curve, which was plotted for each concentration.

### Quantitative PCR assay for miRNA and mRNA

Total RNAs containing miRNAs in cells were prepared using mirVana™ miRNA Isolation Kit (Thermo Fisher Scientific, USA). Real-time quantitative PCR (qPCR) assay was performed on a MiniOpticon™ (Bio-Rad, USA) system using FastStart Universal SYBR Green Master (Roche, USA). With initial denaturation at 95°C for 120 s, amplifications were performed for 40 cycles at 95°C for 5 s and 55°C for 25 s. Primers for qPCR are listed in Table 1.

**Table 1.** Primers used in real-time PCR.

Gene	Forward primer 5′ to 3′	Reverse primer 5′ to 3′
Lin28a	TTGTCTTCTACCCTGCCCTCT	GAACAAGGGATGGAGGGTTTT
Β-actin	CCTGGCACCCAGCACAAT	GGGCCGGACTCGTCATACT
Let-7a	GGTGAGGTAGTAGGTTGTATAGTT	Uni-miR qPCR primer (TaKaRa)
U6	ACGCAAATTCGTGAAGCGTT	Uni-miR qPCR primer (TaKaRa)

### Western blot

Western blot was performed as described previously (13). Briefly, separated proteins in SDS-PAGE were transferred onto polyvinylidene fluoride membranes and were sequentially immune-reacted with specific primary antibodies and secondary antibodies. Antibody-conjunct proteins were quantified using SuperSignal™ West Pico Chemiluminescent Substrate (Thermo Scientific, USA). The primary antibodies cleaved caspase-3 (Cell Signaling Technology (CST, USA)), Bcl-2 (CST), Bax (CST), Lin28a (CST), Sp-1 (CST), HMGA2 (CST), K-ras (CST), Snail (CST), and Zcchc11 (Abcam, UK) were applied after the membranes were blocked in either 5% milk or 5% BSA. Anti-β-actin-peroxidase antibody was obtained from Sigma-Aldrich (USA) and used as an internal reference. The protein content was analyzed using Image Lab 5.1 (Bio-Rad, USA).

### Vector and miRNA transfection

Cells were cultured in 6-well (qPCR and western blot) or 24-well (luciferase assay) plates and were then transfected with plasmids. The Lin28 open reading frame was inserted into the pcDNA3.1(+) vector (Invitrogen, USA) to express Lin28 (pcDNA3.1-Lin28) with Lipofectamine™ 3000 (Invitrogen) in HepG2.2.15 cells. Let-7a inhibitor (2′-O-methyl antisense oligonucleotide) and the non-specific control were commercially synthesized as previously described (14). For western blot assay, cells in 6-well plates with approximately 80% confluency were transfected with 50 nM of let-7a miRNA inhibitor negative control (MOCK) (Thermo Fisher, USA) or 100 nM of inhibitor using Lipofectamine™ 3000 (Invitrogen, USA). Transfection efficiency was validated by qPCR to directly assess the expression of let-7a.

### Transwell invasion assays

The transwell assay was performed using Matrigel-coated (1:5, BD Biosciences) polycarbonate filters (Corning Costar, USA) (15). Cells were subjected to urolithin A for 24 h before use, and the chemical was present throughout the assay. Invasive cells in the lower chamber were stained with crystal violet and visualized using an optical microscope. Cell invasion was quantitated via the absorbance (600 nm) of eluted crystal violet with a microtiter plate reader as described previously (16).

### Statistics

All *in vitro* assessments were performed at least in triplicate. The data are reported as means±SD. Differences between experimental groups were analyzed by Student's *t*-test or one-way analysis of variance (ANOVA), followed by Dunnett's *t*-test for multiple comparisons using SPSS (13.0) statistical program. P-values of less than 0.05 were considered statistically significant.

## Results

### Urolithin A inhibited cell proliferation and induced cytotoxicity in HepG2.2.15 cells

**Figure 1.**
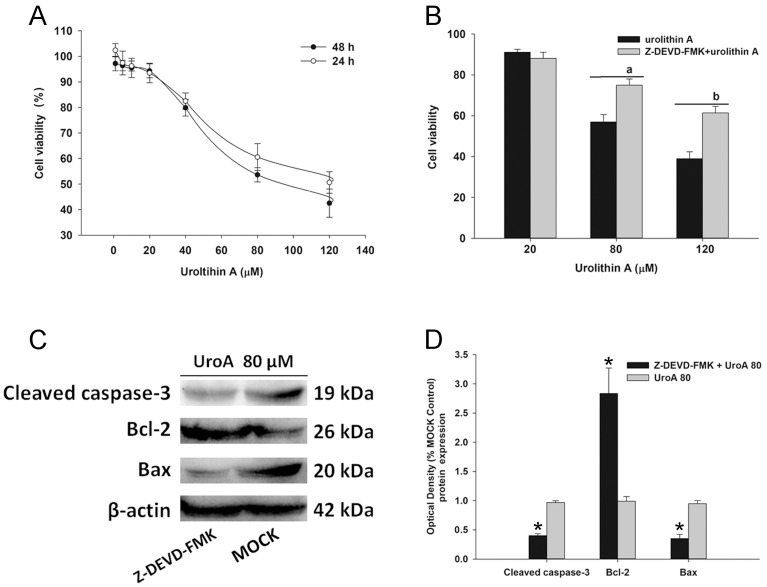
Urolithin A (UroA) suppressed HepG2.2.15 cell proliferation via caspase-3 dependent apoptosis. *A*, HepG2.2.15 cells were administrated with 0–120 µM of urolithin A for 24 or 48 h. Cell viability was assessed by a CCK assay and the data were normalized to normal controls. *B*, The caspase-3 inhibitor Z-DEVD-FMK alleviated urolithin A-induced HepG2.2.15 cell death (80 or 120 µM). The cells were incubated in the absence or presence of Z-DEVD-FMK (20 µM) for 6 h prior to urolithin A treatment, and then incubated for 48 h. Data are reported as means±SD, n=3. ^a,b^P<0.05 *vs* 80 or 120 µM of urolithin A group. *C*, and *D*, After urolithin A (80 µM) administration for 48 h, western blot analysis for the protein expressions of cleaved caspase-3, Bcl-2, and Bax was performed in the absence or presence of Z-DEVD-FMK. Data are reported as means±SD, n=3. *P<0.05 compared to the 80 µM urolithin A group (*t*-test).

In the CCK-8 assay, cells were administrated with urolithin A and B by gradual concentrations (1–120 μM) for 24 and 48 h. As shown in Figure 1A, urolithin A repressed cell proliferation in a dose-dependent manner. It was also found that urolithin A induced acute cytotoxicity from 80–120 μM, compared to the corresponding controls. However, urolithin B (1–120 μM) showed a moderately suppressing effect on HepG2.2.15 cells (Figure S2). Because of this, we chose urolithin A for further investigation. To identify whether urolithin A functions through caspase-3 signaling, cells were incubated in the absence or presence of Z-DEVD-FMK (20 μM) for 6 h before urolithin A treatment. Data showed that pretreatment with Z-DEVD-FMK abolished the inhibiting effect of cell proliferation induced by urolithin A (Figure 1B). Furthermore, we observed that the protein level of cleaved caspase-3 was up-regulated and the Bcl-2/Bax ratio was decreased by urolithin A in HepG2.2.15 cells (Figure 1C and D), suggesting that the suppressing effect of urolithin A on HepG2.2.15 viability could be involved in the activation of caspase-dependent apoptotic signaling.

### Urolithin A altered the expressing pattern of Lin28a/let-7a axis

**Figure 2.**
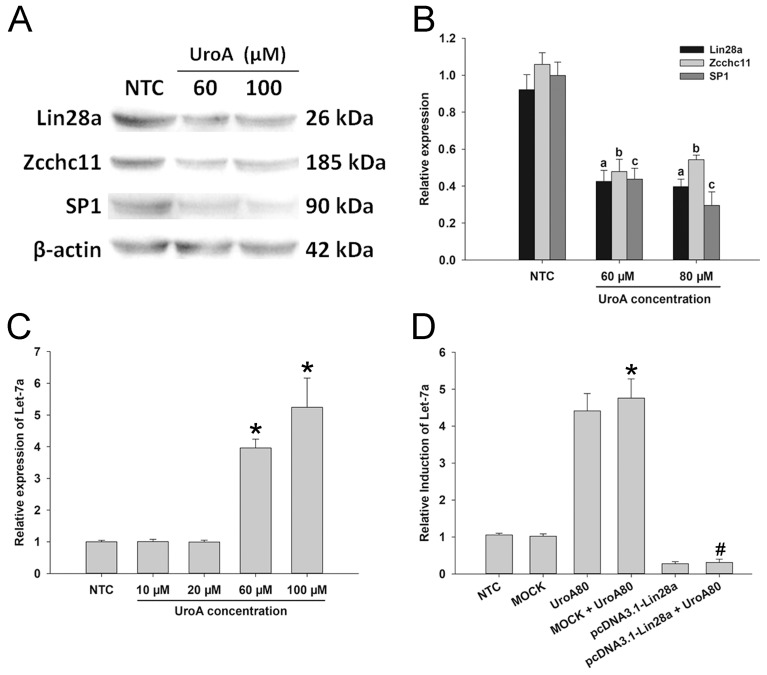
Urolithin A (UroA) regulated the expression of Lin28a/let-7a axis. *A*, The protein expressions of Sp-1, Lin28a, and Zcchc11 were assessed by western blot assay. *B*, Sp-1, Lin28a, and Zcchc11 protein levels were semi-quantified by western blot assay. Data are reported as means±SD normalized to the corresponding β-actin values. ^a,b,c^P<0.05 *vs* the protein expressions of Lin28a, Zcchc11, and Sp-1 in the NTC group, respectively (n=3). *C*, Cells were treated with urolithin A for 48 h. The let-7a expression was quantitated by qPCR. Data are reported as means±SD (n=3). *P<0.05 compared to the NTC group. *D*, The overexpression of Lin28a abolished the effect of urolithin A on let-7a expression (n=3). *P<0.05 compared to the MOCK control. ^#^P<0.05 compared to the MOCK + UroA80 group (ANOVA).

Lin28a, which is a repressor of let-7a by recruiting Zcchc11, could be up-regulated by the HBx protein via Sp-1 in HCC cells. Our data indicated that the protein expressions of Lin28a, Zcchc11, and Sp-1 were suppressed in the urolithin A group, compared to the control group (Figure 2A and B). We also observed that let-7a was up-regulated when the cells were subjected to urolithin A (Figure 2C). To further confirm the effects of urolithin A on the Lin28a/let-7a axis, overexpression of Lin28a (pcDNA3.1-Lin28a) by transient transfection was performed in HepG2.2.15 cells (Figure S3). The results showed that the effects of urolithin A on the expression of let-7a were abolished by Lin28a transfection (Figure 2D), indicating that urolithin A increased the expression of let-7a by targeting Lin28a in HepG2.2.15 cells.

### Urolithin A repressed Lin28a expression and elevated the expression of let-7a in transient HBx-transfected HepG2 cells

**Figure 3.**
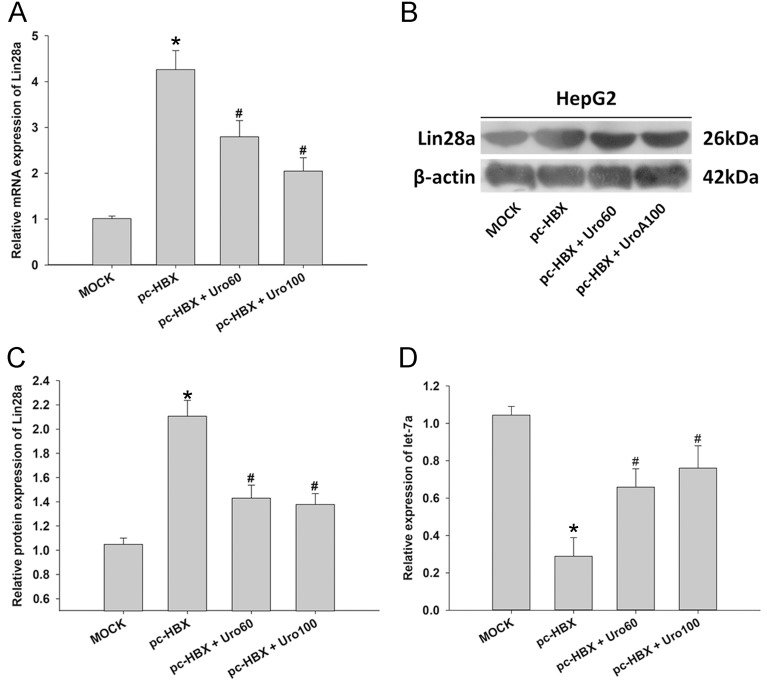
Urolithin A downregulated Lin28a expression and elevated the expression of let-7a in HBX overexpressed hepatocellular carcinoma HepG2 cells. Urolithin A suppressed Lin28a mRNA expression (*A*) and protein expression (*B* and *C*) in HBX-overexpressed HepG2 cells. *D*, Urolithin A elevated the let-7a expression in HBX-overexpressed HepG2 cells (qPCR). *P<0.05 compared to the MOCK control. ^#^P<0.05 compared to the pc-HBX group (ANOVA).

The effect of urolithin A on the Lin28a/let-7a axis was also confirmed in transient HBx-overexpressing HepG2 cells. In this section, HepG2 cells (4×10^5^ cells/well) were seeded in 6-well plates for 24 h and transiently transfected with 2 μg pcDNA-HBx (pc-HBX) plasmids (pcDNA3.1 (-) plasmid with the sequence encoding for the HBx protein; a gift from Prof. Fan Zhu at Wuhan University). After post-transfection for 24 h, cells were incubated with urolithin A (60 and 100 μM) for 48 h. As expected, qPCR assay showed that the elevated mRNA expression of Lin28a in the pcDNA-HBx group was repressed by urolithin A (Figure 3A). To examine whether urolithin A affected Lin28a at the protein level in the HBx-transfected HepG2 cells, western blot analysis was performed; the results demonstrated that Lin28a protein expression was suppressed (Figure 3B and C). Moreover, the HBx-induced let-7a decrease in the pcDNA-HBx group was alleviated by urolithin A treatment (Figure 3D). These data indicated that urolithin A delayed the HBx-induced change of the Lin28a/let-7a axis in HCC cells, further enhancing the findings involved in the effects of urolithin A on Lin28a and let-7a in HepG2.2.15 cells.

### Urolithin A suppressed cell invasion by inhibiting K-ras/HMGA2 signaling in HepG2.2.15 cells

**Figure 4.**
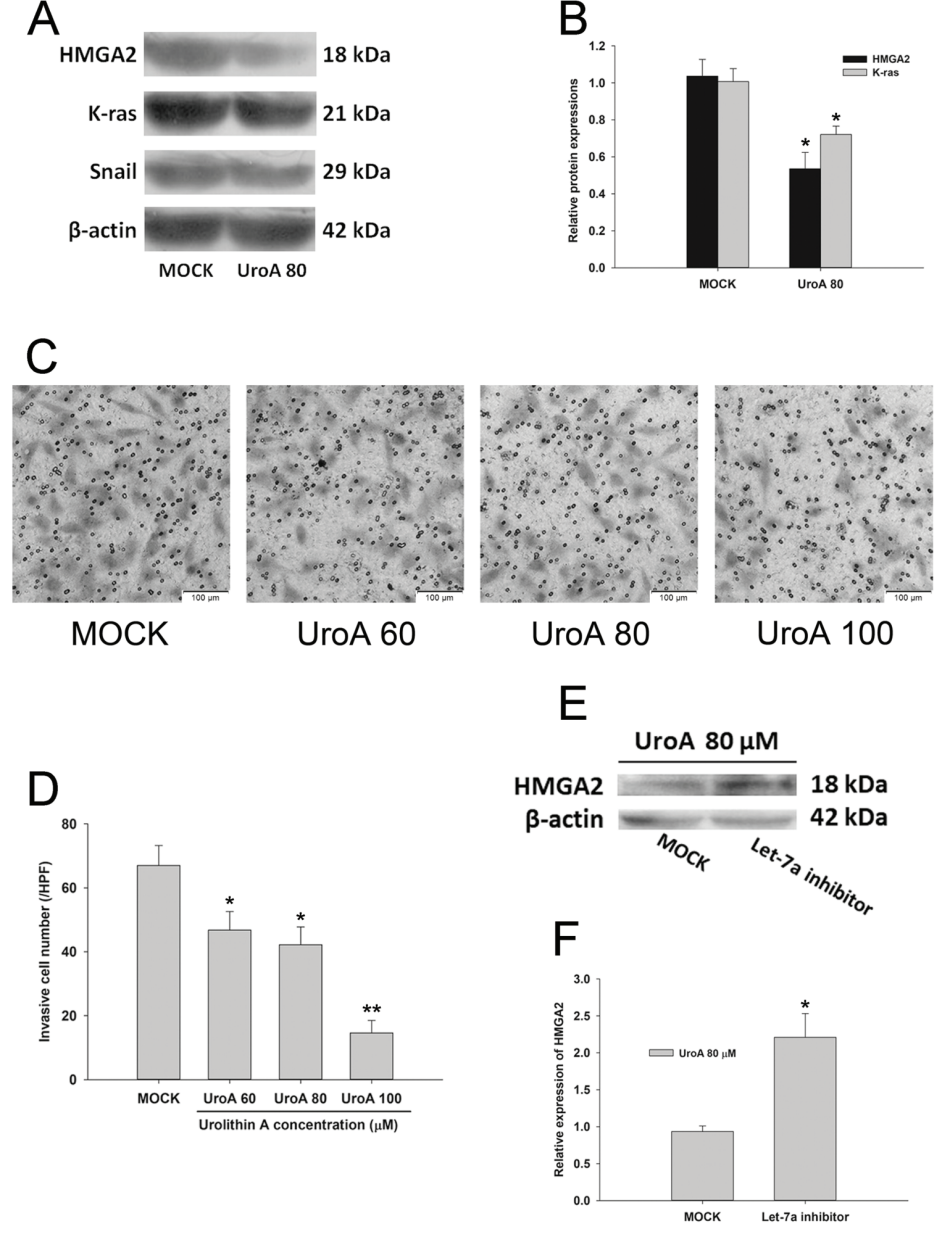
Urolithin A (UroA) suppressed cell invasion by targeting HMGA2/K-ras axis. *A*, Western blotting analysis of HMGA2, K-ras, and Snail in HepG2.2.15 cells treated by urolithin A (80 µM). *B*, HMGA2 and K-ras protein levels quantified by western blot optical analysis. Quantitative data are reported as means±SD (n=3). *C*, Cell invasion was evaluated by a Matrigel invasion assay after 48 h incubation with urolithin A (60, 80, and 100 µM). Magnification bar: 100 µm. The average cell number migrated per high power fields (HPF) is shown in *D*. Quantitative data are reported as means±SD (n=3). *E*, Let-7a inhibitor abolished the suppressing effects of urolithin A on HMGA2 expression by western blot analysis. *F*, Quantitative data from 2 blots shown in *E*. n=3. Data are reported as means±SD. *P<0.05, **P<0.01 compared to the MOCK group (ANOVA).

Since let-7a was increased by urolithin A, we assumed that downstream targets of let-7a could respond to urolithin A exposure. Accompanied by an elevation of let-7a (Figure 2C), the expression of HMGA2, a protein enhancing oncogenic transformation and epithelial-mesenchymal transition, was reduced by urolithin A compared to the MOCK group (Figure 4A and B). The effect of urolithin A on cell invasion was evaluated using a transwell assay. As shown in Figure 4C and D, cells in the control groups exhibited approximately 3 times higher invasion efficiency than cells in the urolithin A (UroA100) group, indicating that urolithin A could suppress cell invasion *in vitro*. To further determine whether let-7a elevation contributed to the suppressing effect of urolithin A on cell invasion, the let-7a inhibitor was transfected for 24 h to abolish the expression of let-7a before urolithin A administration (Figure S4). In Figure 4E, the blockade of let-7a resulted in an increase in HMGA2 protein expression in the western blot assay, compared to the urolithin A group. Moreover, K-ras, which was commonly considered to be a compatible factor of HMGA2 to accelerate cell migration and invasion, was also down-regulated in the urolithin A group (Figure 4A). However, the protein expression of Snail showed no difference between the urolithin A and the MOCK groups. Hence, our data suggested that the regulation of the K-ras/HMGA2 signaling by urolithin A may contribute to the inhibiting effect on cell invasion.

## Discussion

Accepted theory claims that the intestinal metabolism of ETs or EAs to dibenzopyran-6-one derivatives (urolithins) may play an indispensable role in the absorption of ETs. From this perspective, urolithins identified in tissues are important, and their molecular modulation in target cells can be more effective in interpreting the nutritional benefits for human health than that of ETs (17).

Urolithin A is an essential metabolite generated in humans after consumption of EA- and ET-rich food and healthy supplements (18). Cell signaling transduction (EGFR, β-catenin), essential regulators for cell cycle (cyclin D1, c-Myc, p21), and programmed cell death (p53, PARP, caspase-3) could be regulated by urolithins (mainly urolithin A and B) in cancer cells (19). Though the lipogenic gene in hepatoma Huh7 cells and WNT signaling in HepG2 cells are targets of urolithin A or C (20,21), the functions of urolithins in hepatocytes have not been adequately evaluated. Hence, to explore the capacity of urolithin A on HBx-relevant cell invasion, the Lin28a/let-7a axis and Sp-1, a transcriptional factor that could be elevated by HBx, were assumed in the present study to be potential targets of urolithin A in HepG2.2.15 cells.

Sp-1 is an HBx-combinative activator and a member of the Sp/KLF family of transcription factors, and is widely overexpressed in neoplasms, including liver carcinoma (22,23). Based on the anti-proliferative effects of urolithin A in HepG2.2.15 cells, we investigated whether urolithin A could affect the expression of Sp-1. The results revealed that the protein expression level of Sp-1 was repressed by urolithin A treatment. Lin28a, the transcriptional target of Sp-1, which could further elevate the levels of certain cancer-related miRNAs (24), was also suppressed by urolithin A in HepG2.2.15 cells. Therefore, we speculated that suppression of Sp-1 could be responsible for the decrease of Lin28a by urolithin A.

Let-7a functions as a tumor suppressor and is biologically deleted in several cancers, including in HCC patients with HBV infection (25). Moreover, Lin28a suppresses let-7a miRNA biogenesis in tumor cells by recruiting Zcchc11 (TUT4) in the cell cytoplasm to degrading pre-let-7 (26). In our study, urolithin A elevated the let-7a expression, which was delayed by Lin28a overexpression in HepG2.2.15 cells. The similar effects of urolithin A on the Lin28a/let-7a axis were further observed in HBx-overexpressed HepG2 cells. Some evidence has also highlighted that the Zcchc11 down-regulation is Lin28b-independent, and should be restricted to Lin28a-positive carcinomas, such as the T47D breast cancer cells (27). In the present study, the protein expression of Zcchc11 was reduced by urolithin A treatment, suggesting that Zcchc11 inhibition plays a bridging role for the effects of urolithin A on the Lin28a/let-7a axis.

High mobility group AT-hook 2 (HMGA2) is a transcription factor highly expressed in the embryonic stage. The exceptional re-expression of HMGA2 by chromosomal rearrangements at chr12q13-15 in neoplasia of mice or humans, such as HCC and lung cancer, is involved in the let-7 deletion or loss of let-7-binding sites (28–30). In this study, the western blot analysis supported the notion that urolithin A can down-regulate HMGA2 expression and the capacity is let-7a-dependent. We also found that the invasive potential of HepG2.2.15 cells could be repressed by urolithin A treatment. Let-7a also affected oncogenic K-ras expression by binding the 3'-UTR of K-ras. Exogenous let-7a miRNA in HepG2 cells could specifically reduce abundant K-ras expression (31). The protein expression of K-ras was decreased by urolithin A in HepG2.2.15 cells at the repressible concentration in the transwell assay. Therefore, it is possible that the functions of urolithin A in HepG2.2.15 cells may partly match the above-identified proposal involved in let-7a/HMGA2/K-ras signaling.

In the present study, we described the effects of urolithin A in repressing the proliferation and invasion of the HBV-overexpressed HepG2.2.15 HCC cell line. In fact, the virus replication in HBV-infected patients could not be eliminated, but only alleviated with antiviral agents, such as adefovir. The data in this study could be considered an update of our previous data (10) on HepG2 cells because urolithin A has suppressed cell invasion in HepG2.2.15 cells. In other words, our findings are not only involved in the targets of carcinogenesis but are also based on the initial observation of the HBx-interactional cancerous transcriptional factor, Sp-1. Therefore, our data demonstrated that urolithin A suppressed the HepG2.2.15 cell proliferation and invasion via regulating the Lin28a/let-7a axis and EMT-involved targets, such as HMGA2 and K-ras.

## Supplementary Material

Click here to view [pdf]
